# Memories of Visual Events Can Be Formed Without Specific Spatial Coordinates

**DOI:** 10.5334/joc.104

**Published:** 2020-06-08

**Authors:** Shekoofeh Hedayati, Brad Wyble

**Affiliations:** 1 Department of Psychology, The Pennsylvania State University, University Park, PA, US

**Keywords:** visual binding, spatial location, visual search, awareness

## Abstract

To what extent does specific spatiotopic location accompany the remembered representation of a visual event? Feature integration theory suggests that identifying a multi-feature object requires focusing on its spatial location to integrate those features. Moreover, single unit data from anterior ventral stream neurons that fire preferentially to complex objects indicates that they have retinotopic receptive fields. It can, therefore, be predicted that location information of features of a complex stimulus is inherent in the memory of a perceived visual stimulus’ representation. To evaluate this prediction, we presented participants with a brief array of characters with instructions to identify and locate the solitary letter among a set of digits. Surprisingly, analysis of trials in which the target identity was accurately reported indicated that in more than 15% of trials (i.e., in Experiments 2b & 2c) participants were almost completely uninformed about the location of the letter that they had just identified. Further analysis showed that there were two main sources of these location errors; misbinding the target to the distractors’ locations and extremely poor spatial representation of the target’s location to an extent that was indistinguishable from guessing. The latter finding indicates that consciously accessible representations of visual events can form despite being untethered to robust and spatially-specific representations, implying that the specific location was either not quite encoded into working memory, or was rapidly forgotten. However, when the target was marked by a single feature (color), there was no evidence of remembering the target identity without remembering its location even with strong masking.

## Introduction

Our daily visual experiences of identifying objects often involve selecting objects located amongst other items. For example, recognizing a coffee cup on a desk often coincides with knowing at least its approximate location (e.g. it is somewhere on a desk). Therefore, the ability of our visual system to select relevant information in the cluttered world and link it to its location is a crucial aspect of perceiving the environment. Moreover, as the selection process becomes more difficult (e.g. expending more effort to find the coffee cup on the desk, because the desk is more cluttered with the presence of other objects), the linkage of objects to their locations is expected to be strengthened due to requirements of marshalling attention at the item’s location prior to identification.

In this regard, long-standing theories of spotlights (Eriksen & Hoffman, 1972) and feature integration (FIT; Treisman & Gelade, 1980) highlighted the critical role of attending to the target’s location in demanding attentional tasks, whereas in simpler visual searches such as a single feature task (e.g. finding a red square amongst back squares) identification can be accomplished without spatial focus of attention. In other words, for difficult visual searches (e.g. a conjunction of features), attention should be deployed to the target’s location in order for the item to be identified (Johnston & Pashler, 1990; Treisman & Gelade, 1980). Consistent with this idea, Evans and Treisman (2005) found that although targets in a natural scene could be *detected* without being successfully identified or localized, a further binding process of attention was required to identify or locate the target. Furthermore, there is empirical support that location information always accompanies reports of visual percepts (Carrigan et al., 2019), even in cases where participants are not expecting location to be a task-relevant attribute (Chen & Wyble, 2015). Thus, it seems plausible to think that reporting a target’s identity defined by a specific pattern of features, such as choosing a letter from a set of digits, requires focused attention to be deployed to the target’s location.

Outside of visual search, when subjects are asked to remember a list of letters presented at different spatial locations, there are findings of bindings between spatial locations and letter identity. Guérard et al. (2009) found that phonological and spatial information are bound such that phonological similarity disrupts spatial memory in addition to letter memory. This is particularly notable because the letter-location bindings were entirely task irrelevant (i.e., subjects were never asked to indicate which letters had appeared at which locations). Moreover, the phonological interference in spatial report was found even when the retrieval of letters was not necessary for the entire experiment (Guérard et al. 2013).

In addition to these theories derived from human behavior, electrophysiology studies on primates revealed that neurons with form-specific preference in anterior portions of the ventral visual pathway are also strongly sensitive to retinal position. This finding suggests that form and location information are already bound together when these neurons are activated by their preferred stimulus (Dicarlo & Maunsell, 2003). Assuming that such neurons are involved in the identification of a complex stimulus, these findings further reinforce the hypothesis that identification of such a stimulus should coincide with the ability to report its location, to at least a moderate degree of precision. Consistent with this were findings from an fMRI study that indicated significant sensitivity of lateral occipital cortex (i.e., anterior to V3d andV3a/b and posterior to MT) to object’s retinal position. Neural activations elicited by retinotopic position were found to be even stronger than activations related to object categories (Sayres & Grill-Spector, 2008).

Moreover, theories of the neural mechanisms underlying conscious awareness of visual events typically posit that the early levels of the visual hierarchy play a key role in supporting a high-level visual percept (Lamme, 2006). Thus, awareness of a complex patterned stimulus, such as a letter, to a degree that allows explicit report of its identity, might be thought to therefore include active representations in early levels of processing that are highly location specific. This theory of early-level active representations does not necessarily mean that the spatial location is *required* to form the percept, but rather it implies that the location information is expected to be included within the percept, since early visual areas are highly location specific.

This collection of accounts and findings provide convergent support for the prediction that some degree of location information should always be part of an explicitly reportable representation of a visual event, particularly when identification requires a complex pattern discrimination.

Based on these findings and theories, we predicted that whenever people report the identity of a letter that is presented among digits, they will always be capable of reporting its location to a moderate degree of precision. However, this was not the case. On a proportion of trials, subjects could recall the identity of a single letter in a brief display surrounded by digit distractors without being able to report its location within even the general vicinity of the target, despite the fact that the location probe happened a few seconds after the array was viewed. The failure to report location was present even after applying correction for identity guessing and an analysis that adjusted for location swaps with distractors. This was a striking observation that violated our intuitions, as it suggested that location information was not always inherent in the mental representation of such items after they had been identified. A final experiment demonstrated that when target letters were indicated by a simple pop-out color, it was always possible to report the target’s location even when identification accuracy was very low due to strong masking. Collectively, these results run counter to the typical understanding of visual attention, which would posit that increasingly complex visual discriminations are associated with an increased reliance on spatial information. These results have important implications for theoretical frameworks regarding the nature of visual percepts. It is important to mention that our primary interest is in objective measures of location and identity information, rather than subjective or meta cognitive information. Therefore, no subjective report of awareness (e.g. asking about the level of confidence) was gathered in the experiments.

## Experiment 1a

In the first experiment, we tested whether participants could report the location of a letter that they have identified.

### Method

#### Participants

Twenty participants from the Pennsylvania State University undergraduate psychology subject pool received credits in exchange for participation. The sample size was informed by a power analysis using permutation-based bootstrapping of 5, 10 and 20 subjects from an initial pilot study (similar to Experiment 1a). This analysis revealed that a sample size of 20 participants was sufficient to show reliable evidence that location errors occur with a non-zero probability. During the power analysis, 5,10 or 20 subjects with their estimated location errors (see formula 1 in supplementals) were sampled randomly with replacement from a set of 20 subjects. For each simulated subject set, subjects were selected randomly with replacement 100 times to provide a bootstrapped estimate of the confidence interval for the location errors. The confidence interval was computed by averaging the 2.5% lower and upper bounds across those 100 bootstraps. This process was repeated for 100 times to generate a set of confidence intervals. This bootstrap indicated that with 20 subjects the confidence intervals did not include 0, 100% of the time.

Participants were instructed in English and reported normal or corrected-to-normal visual acuity. The experimental design was approved by the IRB at The Pennsylvania State University.

#### Apparatus

Participants were positioned on a chinrest 60 cm from a 17-inch, 1024 × 768 CRT monitor. Experiments were performed using Psychtoolbox 3 (Kleiner et al. 2007) in MATLAB (build 2012b) operating on windows XP. Responses were made using a computer keyboard or a mouse depending on the task.

#### Stimuli and procedure

**Figure 1:**
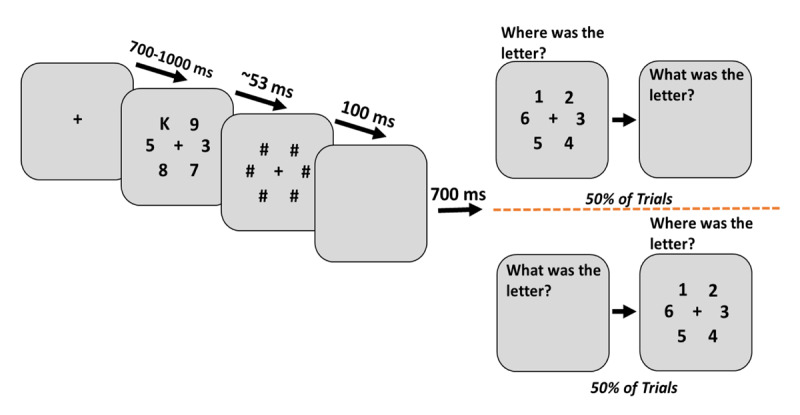
A trial example for experiment 1a. A black fixation cross was presented for 700–1000 ms in the center of a gray background. Stimuli consisting of a letter and five digits were shown for roughly 53 ms and then masked for 100 ms. Participants were asked to report the letter and its location during each trial with the two questions in random order.

As shown in Figure 1, a fixation cross appeared in the center of a gray (127,127,127 in RGB) screen at the beginning of each of 96 trials. After a varied time interval (700–1000 ms), stimuli (height = .86°) consisting of one letter chosen randomly from a set of capitalized letters (R, L, C, P, F, K, B, G, Y, V, H, X, T, J, D) and five digits (2–9) evenly spaced on a hypothetical circle (diameter = 4.54° visual angle) were presented for ~ 53 ms and then followed by hash-mark masks (height = 1.05°) for 100 ms. Participants were asked to report the identity of the letter by typing one of the 26 letters on the keyboard and the location of the letter by pressing a number from 1 to 6 that corresponded to the target location. The task did not impose speed pressure and subjects were allowed to use backspace to correct their responses. The order of the questions was randomized and counterbalanced during the experiment. Each experiment began with 7 slow trials that became progressively faster, with stimulus duration moving from 1 sec to 53 ms. These slow trials were excluded from the analysis.

### Analysis

In the final analysis, we measured accuracy and determined statistical significance using 95% confidence intervals estimated by a bootstrapping method with 500 resamples at the subject level. That is, location error for each subject was estimated using formula (1) described in supplementals. Then, 20 subjects were sampled randomly with replacement 500 times to provide a bootstrapped estimate. The confidence interval was estimated by averaging the 2.5% lower and upper bounds of estimated location errors over these 500 bootstraps.

### Results

**Figure 2:**
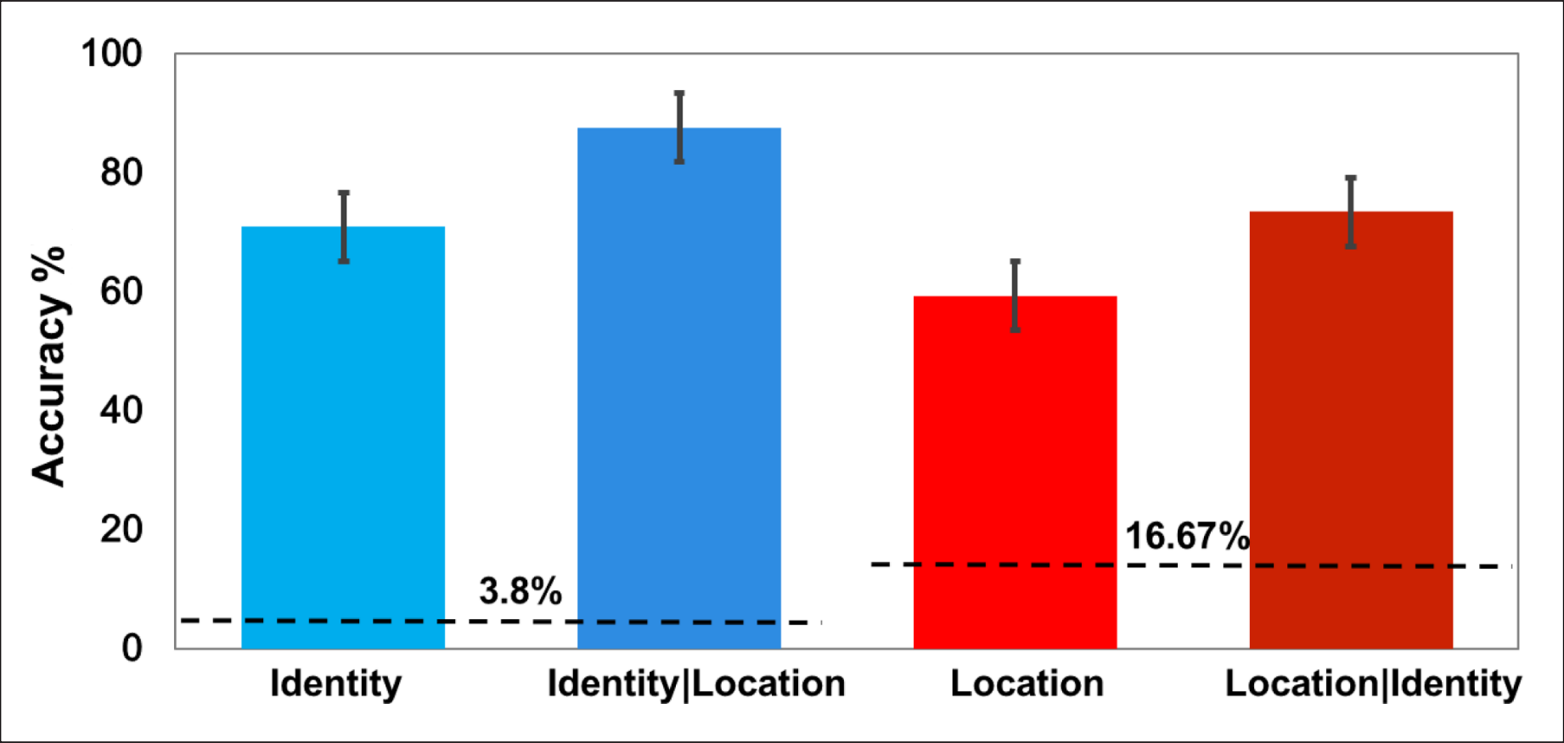
The results from Experiment 1a, before correcting for guesses. The first and third bars show the accuracy of identity and location respectively. The second bar depicts identity accuracy for trials in which participants correctly reported the target location. The last bar represents location accuracy for trials in which the target was correctly identified. Error bars represent standard errors and the horizontal dashed lines represent chance levels.

Accuracy for location report was 58% (chance was 16.67%) and for identity report was 70% (chance was 3.8%), indicating that the task was challenging, but both were far above chance levels (Figure 2). Moreover, conditional probability of identity accuracy when location report was accurate was 86%, while location accuracy when identity was reported correctly was 71%. Thus, on 20% of all the trials, participants could report the identity of the letter without being able to report its location from among 6 locations.

Additional calculations, described in the supplementary materials, corrected for cases where the *identity* was presumably reported correctly with a lucky guess. From trials in which participants identified the letter successfully (observed value of 70%, of which 1.7% are likely to be lucky guesses, thus the estimate of true identification = 68.8%) they reported the location accurately an estimated 63.5% of the time after further correction for *location* guesses. Thus, from those trials with correct letter report, participants reported the wrong location an estimated 36.5% of the time, 95% bootstrapped CI[25%, 45%].

As an exploratory analysis, to determine if there was a *coarse-grained* memory of target location, we measured the frequency of responses for the two locations adjacent to the target and their two opposite locations (i.e., In the trial depicted in Figure 1, we compared reports at the two locations adjacent to the letter K, to the two positions containing the 8 and the 3; the location directly opposite the K was ignored). Surprisingly, the data showed that there was only a very small advantage for the neighboring vs the distant locations and both were substantially lower than the responses for the actual position of the target; accurate location: 71%, the two adjacent locations: 14% and the two distant locations: 10.5%. This means that for reports at these four locations —two neighboring the target and two on the other side of the array— participants were reporting on adjacent locations, only 57% of the time (14/(14+10.5) = ~ .57) with chance level to be 50%.

## Experiment 1b

To test if this result would replicate when stimuli are presented farther in the visual periphery, thus increasing center-center spacing, we repeated experiment 1a with increased eccentricity.

### Method

This experiment was identical to experiment 1a, except that the eccentricity was doubled (9.1° visual angle). A new set of 20 students was recruited.

### Results

**Figure 3:**
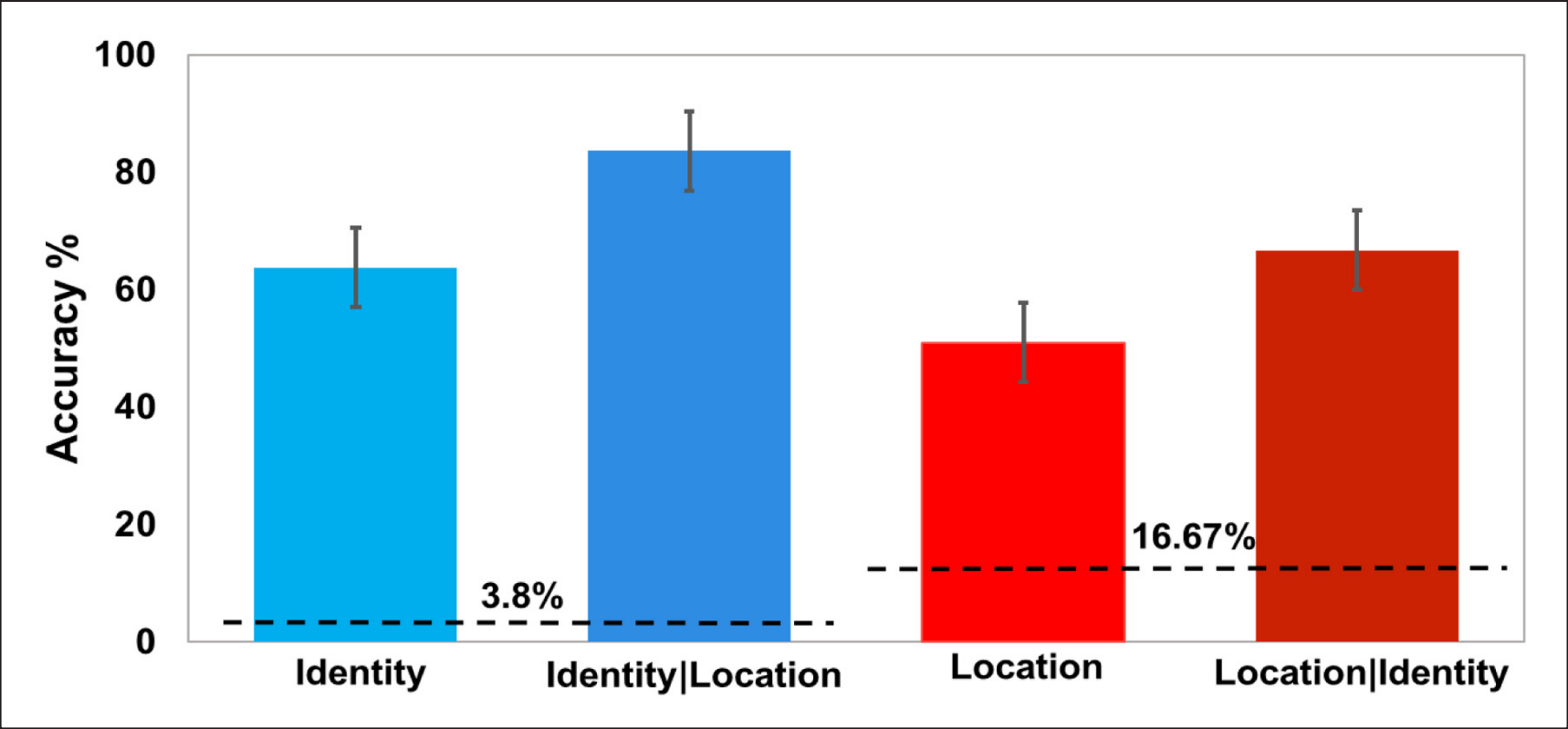
The results from Experiment 1b, before correcting for guesses. Bars represent the same parameters as in Figure 2.

As represented in Figure 3 and Table 1, the results were consistent with the previous experiment. Identity accuracy was 62.7% and location accuracy was 49%. Conditional analyses revealed that identity accuracy was 73% when location was known, and location accuracy was 64% when identity was known.

**Table 1:** Performance on identity and location in trials where identity had been reported successfully. The last column (numbers in bold) represents location report error rate in successful identity reports after correction for all guesses.

Experiments	Identity accuracy	Location accuracy given correct identity			True location errors

	Observed	Observed	After correction for location guessing	After correction for location and identity guessing	

1a	70%	71%	65.2%	63.5%	**36.5%**
1b	62.7%	64.3%	56.8%	54.5%	**45.5%**

From trials in which participants identified the target letter correctly (observed value of 62.7%, of which 2.3% are likely to be lucky guesses, estimated true value = 61.21%) they reported the correct location only an estimated of 54.5% of the time after location guessing correction. This means that participants missed the location of target letters that they correctly identified 45.5% of the time, 95% bootstrapped CI[38%, 48%].

Measuring coarse-grained location information by comparing reports of neighboring and distal locations indicated that, similar to the results of experiment 1a, that was only a very small advantage for the adjacent locations: correct: 64.3%, adjacent: 16% and distant: 14%. Repeating the computation from Experiment 1a revealed that incorrect location reports were biased towards the correct half of the display only 53% of the time.

### Discussion

Experiments 1a and 1b provided insight about the concurrence of reportable location and identity information for the target items, however, as location was constrained to six evenly spaced locations, it could not be conclusively determined if errors reflected a coarsely grained location representation or a more severe lack of location information (i.e., uninformed responses). To define these terms for our purposes, precise location responses would be largely confined to the quarter of the notional circle centered at the target, and coarse-grained responses would be largely confined to the half of the circle that contained the target, and uninformed responses would be essentially uninformed by the target’s location.^1^

Our analysis of experiments 1a and 1b suggested that location reports were either precise or relatively uniform with little evidence for a coarse-grained location representation that spans half of the display, since adjacent locations were selected only slightly more often than the distant locations. The uniformity of the location errors also implied that probably some proportion of errors resulted from the lack of location information rather than misbinding to distractors’ location. In other words, misbinding to locations is expected to occur substantially more frequently for the neighboring locations than distant locations (Johnston & Pashler, 1990). Since we observed 16% of responses to be made at adjacent locations, and 14% at distant locations in Experiment 1b, we conjectured that not all the location errors are due to misbinding, but rather there are some errors resulting from a near complete lack of location information. However, this paradigm did not afford us an easy way to statistically verify this conjecture. We tested this hypothesis more directly in Experiment 2, where we directly measured the quality of response errors using a continuous paradigm.

To more directly measure the quality of location report errors, we devised Experiment 2. This experiment will allow us to determine 1) the precision of location reports relative to true position of the identified target. 2) Disentangle the trials in which the subjects misbound the target to the distractors’ location from those in which the target was not bound to any specific location.

## Experiment 2a

To more directly measure the quality of location responses, the stimulus locations and response method were modified to a continuous form in which stimuli could occur at any location on a circle and participants chose a specific location with a mouse click.

### Method

**Figure 4:**
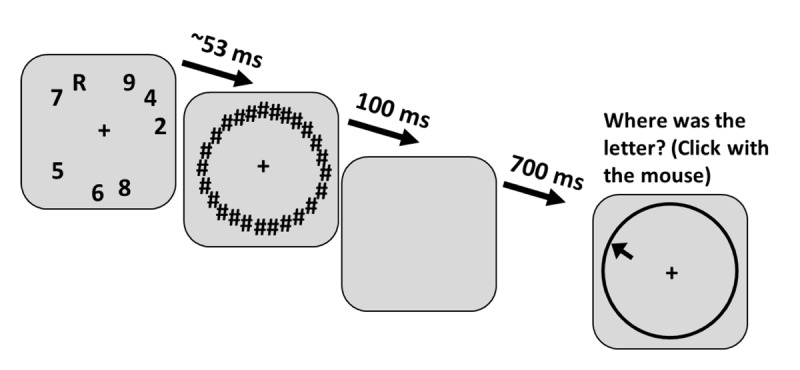
Experiment 2a trial example. Participants reported both location and identity in each trial in randomly selected order, but the question of identity has not been included in the figure. There were 60 hash-marks in the actual trial. The black arrow indicates the mouse pointer that had to be moved to the circle to complete a response.

Keeping most parameters identical to Experiment 1b, 8 stimuli were presented at random locations on an invisible circle and masked (Figure 4) in 144 trials. A minimum distance between locations (1° of visual angle) prevented stimuli from being placed too closely. Participants reported the letter identity as before but now reported location by moving the mouse curser and clicking on a circle to indicate the precise angle of the target. Furthermore, to prevent participants from using mask locations (masks remained on the screen for 100 ms after target offset) to anchor their memory of the target location, we masked uniformly around the circle.

### Results

**Figure 5:**
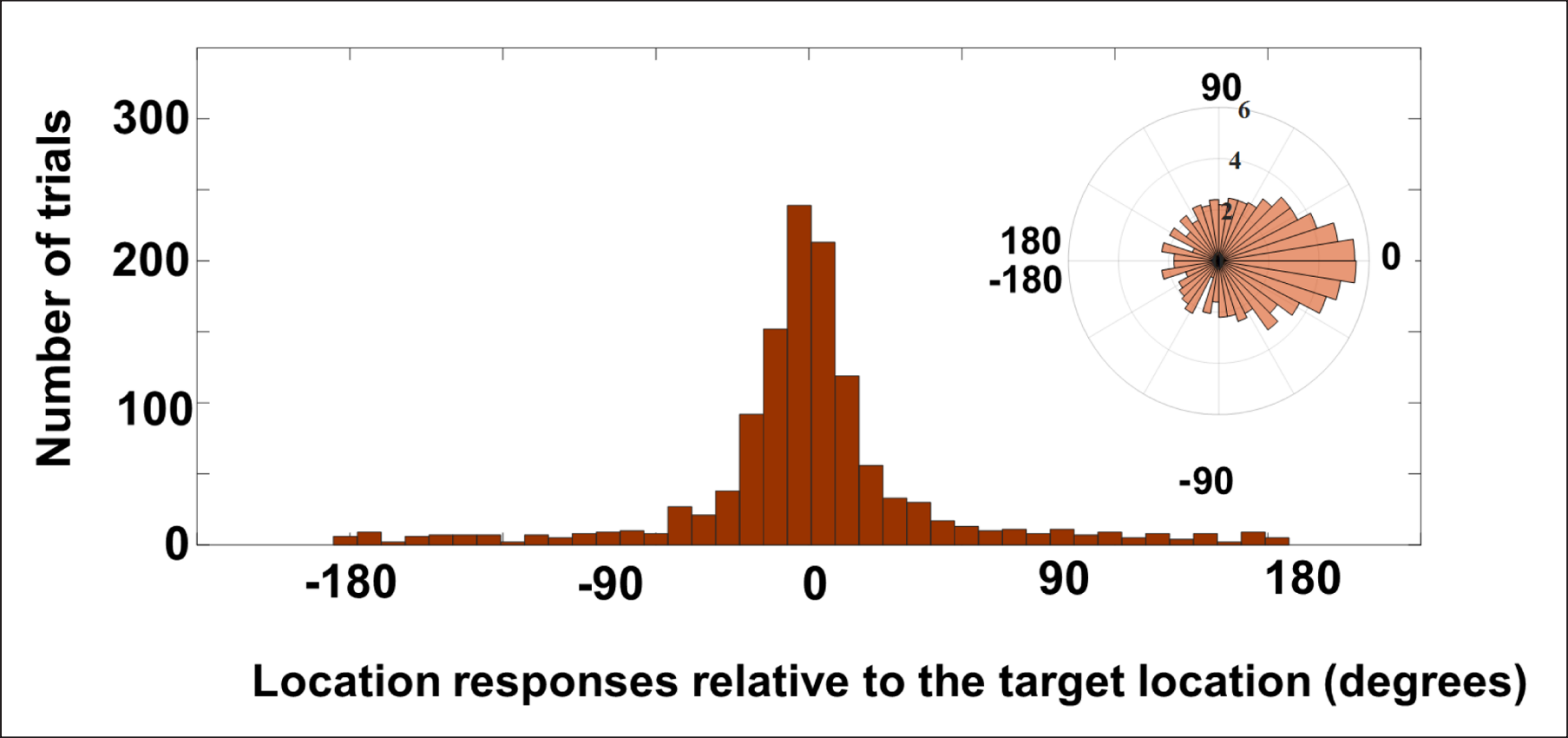
Location report data of Experiment 2a for all correctly identified letters. The polar plot has been log-transformed for the ease of visualizing low-N bins.

Figure 5 illustrates the spatial distribution of location reports relative to the target’s true location for correctly identified letters (i.e., identity accuracy of 45.5%). This distribution is characterized by a clear middle peak, and what looks like a uniform component. Probabilistic mixture modeling (Bays et al., 2009; Zhang & Luck 2008) was used to characterize the location reports as distinct components. A three parameter mixture model consisting of a Gaussian distribution centered at the target location, Gaussian distributions centered at the distractors’ locations and a uniform distribution corresponding to locations that are presumably not available in working memory was used. In the analysis, the K parameter represents precision (i.e., higher precision corresponds to higher K value and lower standard deviation (SD)), NT represents the probability of responding to distractors’ locations and pU represents the probability of responding with spatial accuracy so poorly that it approaches a uniform distribution relative to the target’s location.

### Analysis

Similar to the Experiment 1 analysis, statistical significance was determined using 95% confidence intervals estimated by a bootstrapping method with 500 resamples from correct identity trials. For each of the 500 bootstraps, the set of trials with correct identity report were sampled randomly with replacement X times (where X was the number of trials in which the target identity was correctly reported). Each of these 500 bootstraps were then used to compute the mixture model parameters (i.e., K, NT and pU). The 95% confidence intervals were estimated by averaging the 2.5% lower and upper bounds for each parameter across the 500 repetitions.

To include identity guessing correction, a paired samples t-test was carried out to compare the identity guessing and the likelihood of uniform location responses (pU) for each Experiment. In addition, we conducted Bayesian analysis to determine Bayes factors between identity guessing and pU using JASP 0.11.1.0 software (JASP team, 2019). To interpret Bayes factors, BF_10_ = 1 indicates no evidence in favor of any hypothesis, 1 < BF_10_ < 3 indicates weak evidence in favor of the alternative hypothesis (H1; pU > identity guessing), 3 < BF_10_ < 10 indicates moderate evidence in favor of H1, and 10 < BF_10_ < 30 indicates strong evidence in favor of H1 (Jeffreys, 1961).

#### Mixture model analysis

In the analysis of Experiment 2a, the precision (k) was 12.37 (corresponding to one SD of 16.16° of the 360° circle), CI[10.21, 15.46], nevertheless, this is a reasonably precise memory of target location with nearly all of the central mass within less than 90 degrees. The probability of responding to distractors’ locations (NT) was 13.82%, CI[8.81, 18.82] and the probability of uniform responses (pU) was 10.46%., CI[5.09, 16.01]. Thus, the estimate of responses indicated that after eliminating likely responses that were misbound to distractor locations, subjects still reported locations with such variability as to be indistinguishable from random guessing.

#### Identity guesses correction

On some trials, subjects may have not seen the target and reported it correctly by chance. Such responses would inflate the estimate of pU and must be corrected for. Lucky identity guesses for *each subject* were estimated by the same formula described in the supplemental. Individual estimation of the guesses allowed us to perform a paired-samples t-test between each subject’s identity guesses and their estimated pU. In essence, this tested whether the estimated proportion of responses in the pU component of the mixture model was larger than the estimate of lucky identity guesses.

Note that this correction and test is the most conservative test that could be applied since it assumes that 100% of the identity guesses should count against the pU responses. In fact, it is likely that some lucky guesses of identity would have been associated with location reports centered at one of the 8 items and therefore would have been counted among the target and NT distributions by the mixture model. Since we have no way to estimate that proportion, we adopt the conservative approach of assuming that all lucky guesses were from pU.

Finding a difference here would suggest that the proportion of largely uninformed location reports was above 0% for trials in which the identity was reported. After applying a paired-samples t-test, the results revealed a significant difference between identity guessing (M = 6.2%, SD = 4.82%) and pU (M = 13.94%, SD = 16.48%); t(19) = –2.38, *p* < 0.05, *BF*_10_ = 4.35 indicating that at least some proportion of location errors can be attributed to an almost complete lack of location information at the time of the report.^2^ ^3^ Note that it is difficult to be sure whether these inaccurate responses are truly guessing, or extremely imprecise location estimates (Pratte, 2018; Schurgin et al. 2018).

## Experiment 2b

This experiment is a replication of experiment 2a with an increased number of stimuli (i.e., 10 instead of 8) and 140 trials to see if the same results will be obtained. Also, a new set of 20 subjects were recruited.

### Mixture model analysis

**Figure 6:**
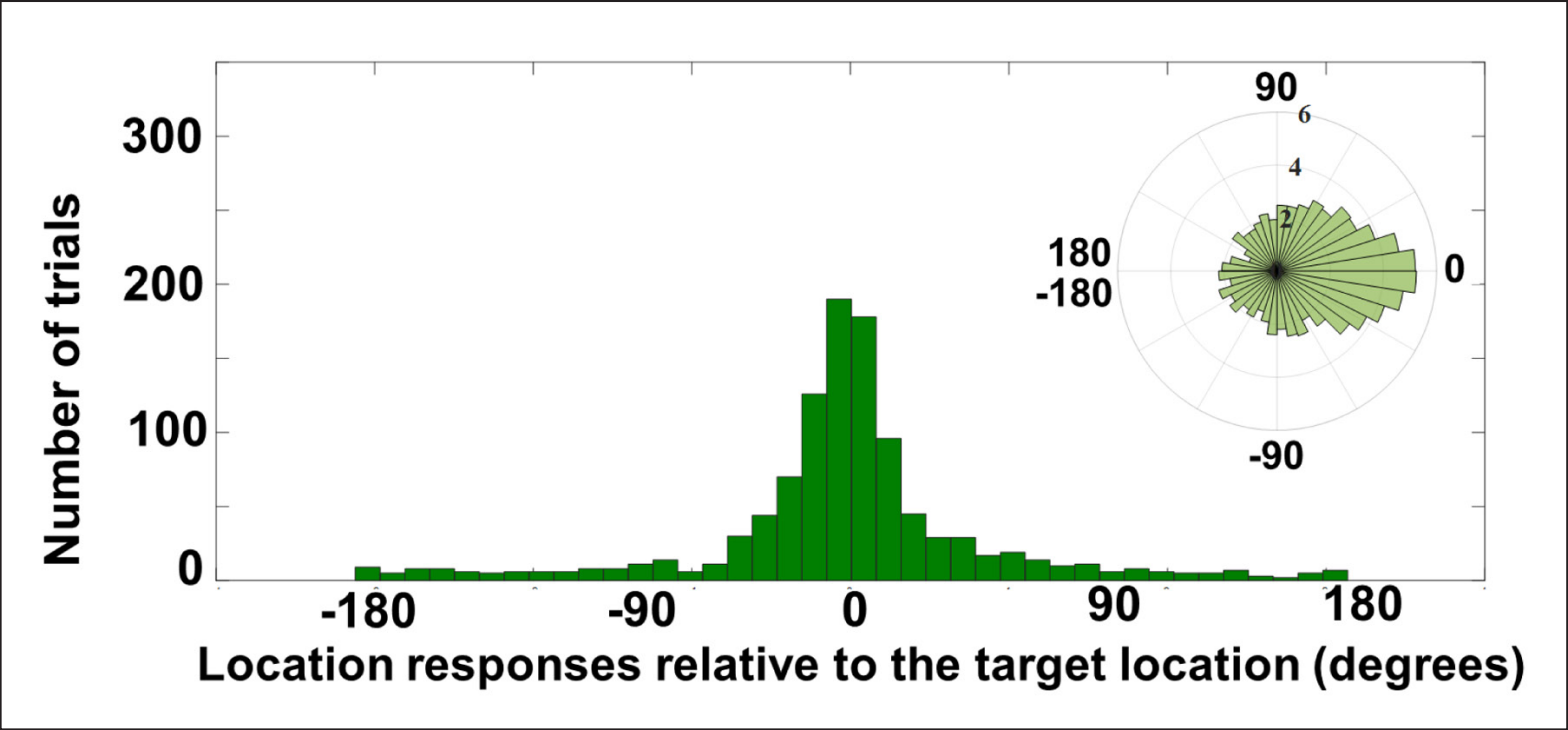
Location report data of Experiment 2b for all correctly identified letters. The polar plot was log-transformed for the ease of visualizing low-N bins.

Figure 6 illustrates the spatial distribution of location reports relative to the target’s true location for correctly identified letters (i.e., identity accuracy of 40.9%). There were 1,079 identity-correct trials. The mixture model analysis resulted in the K value of 11.78 (corresponding to one SD of 17.07° of the 360° circle), CI[9.86 15.58]. Similar to the case of 8 stimuli, the memory of the target location was reasonably precise. The NT component was 13.04%, CI[5.02, 21.26] and the pU component was 15.46%, CI[6.03, 24.09].

### Identity guesses correction

Paired-samples t-test indicated significant difference between identity guesses (M = 6.4%, SD = 3.58%) and estimated pU (M = 17.6%, SD = 17.49%); t(18) = –2.95, *p* < 0.01, *BF*_10_ = 11.75 which was suggestive of participants’ nearly uninformed memory of the target location in some of the trials for which the letter was correctly identified. Note that one subject’s data that had a very low identity accuracy (11%, with 40% overall mean accuracy across subjects) was excluded from the analysis by the model (i.e., degrees of freedom was 18 instead of 19), due to the failure of the mixture model to estimate any parameters for that subject.

## Experiment 2c (replication)

This experiment was a replication of experiment 2b with a new set of 60 subjects to validate the previous results.

### Method

This experiment is identical to Experiment 2b conducted on 60 new subjects.

#### Mixture model analysis

**Figure 7:**
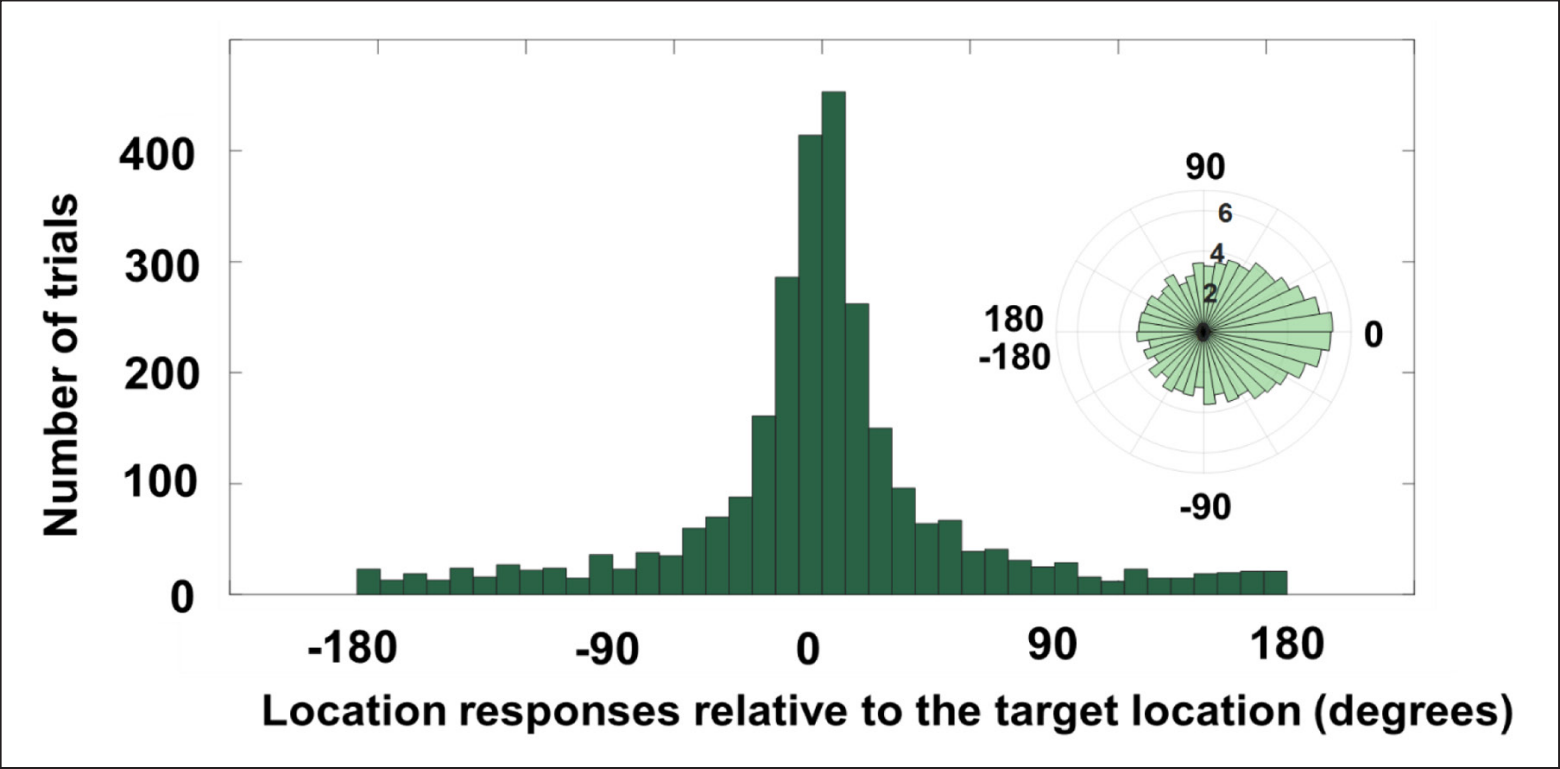
Location report data of Experiment 2c for all correctly identified letters. The polar plot was log-transformed for the ease of visualizing low-N bins.

The results have been depicted in Figure 7 for correctly identified letters (i.e., identity accuracy of 35.4%). There were 2, 826 identity-correct trials. The K value for correctly reported identities was 10.22 (SD = 18.4° from the 360° circle), CI[8.29, 12.36], the NT was 16.57%, CI[11.5, 24.2] and the pU was 16.46%, CI[9, 23.3].

#### Identity guesses correction

The results were consistent with previous experiments. Paired-samples t-test indicated significant difference between identity guesses (M = 9.2%, SD = 6.95%) and estimated pU (M = 16.43%, SD = 19.66%); t(58) = –2.86, *p* < 0.01, *BF*_10_ = 11.14.

## Experiment 3

So far, it has been observed that even approximate location information is not always available in the mental representation of an identified letter. We were interested to know if the same results would be obtained when a single feature (e.g. color) marks the target and its location. To address this, the paradigm was changed so that all of the stimuli were letters, and the target letter was red in color.

### Method

**Figure 8:**
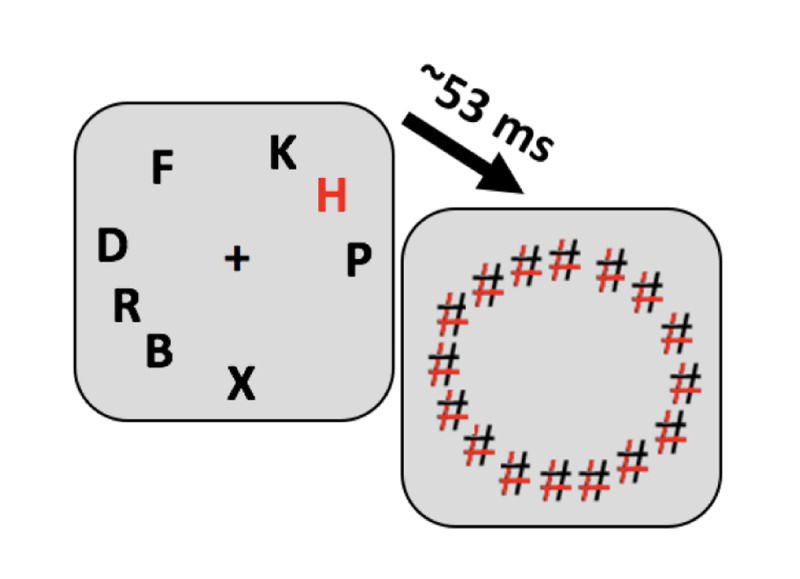
A trial example of Experiment 3 (There were 34 hash marks in the actual display). The response display is similar to Experiment 2.

This experiment is similar to experiment 2a with the following changes: Stimuli were one red letter (target) and seven black letters (distractors). Masks were hash marks comprising two black and two red lines (Figure 8). Participants were instructed to report the identity and location of the red letter.

#### Mixture model analysis

**Figure 9:**
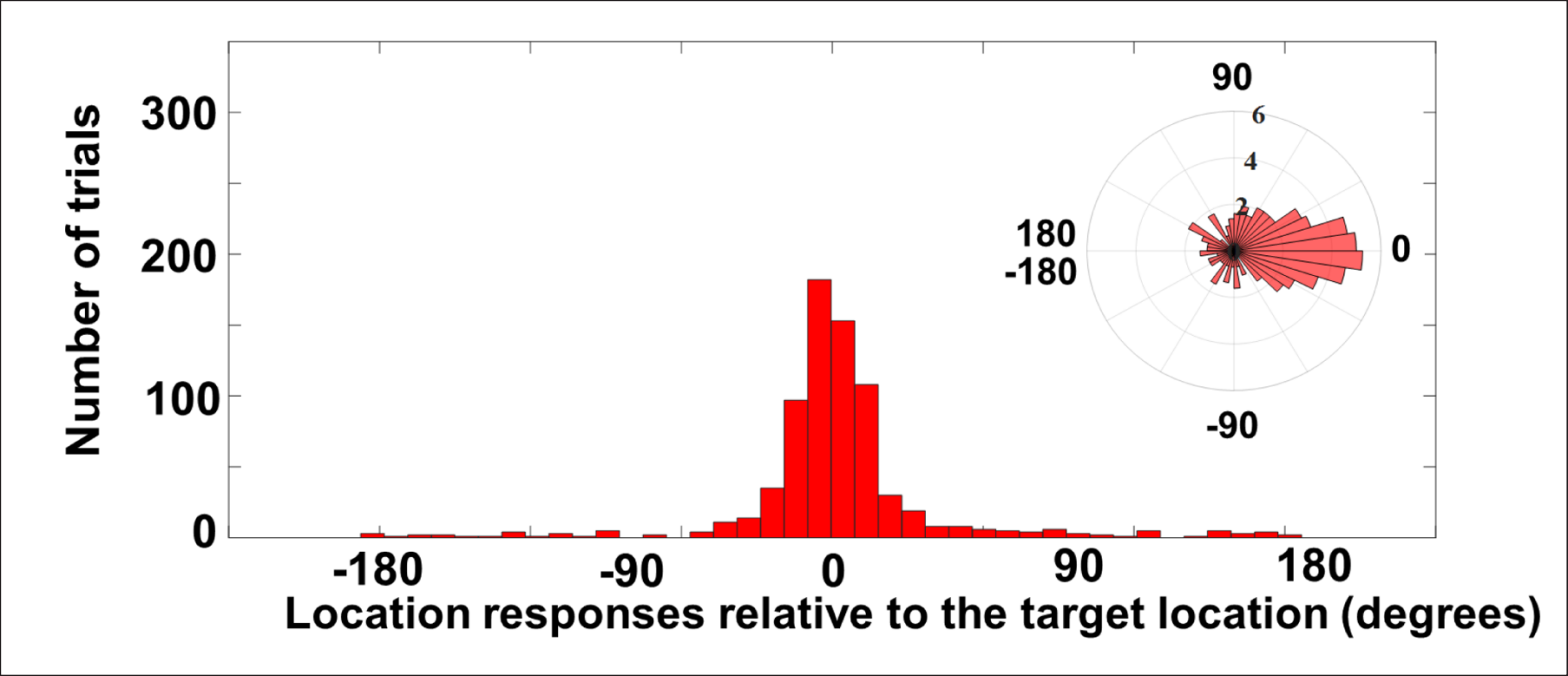
Location report data of Experiment 3 for all correctly identified letters. The polar plot was log-transformed for the ease of visualizing low-N bins.

The results have been shown in Figure 9 for correctly identified letters (i.e. identity accuracy of 27%). There were 742 identity-correct trials. In the mixture model analysis for location reports, the K value was 19.79 (SD = 13.04 ° from the 360° circle), CI[16.5, 24.41], NT was 5.62%, CI[.7, 9] and the pU was 9.96%, CI[4.8, 15.3] for correctly identified letters.

#### Identity guesses correction

There was no significant difference between identity guesses (M = 15.44%, SD = 12.91%) and estimated pU (M = 14.2%, SD = 24.63%); t(18) = .28, *p* = 0.78, *BF*_10_ = 0.19 with the Bayes factor indicating moderate evidence in favor of the null hypothesis. Therefore, there was no evidence that subjects were unaware of the location of the identified letter, as the estimated proportion of pU responses for location could be entirely due to guessing the identity. This result shows that the failure of location report is not a consequence of the rapid presentation format as presentation duration was constant (~53 ms) across all experiments.

## General Discussion

The first experiments (1a & 1b) suggested that participants reported the wrong location of a correctly identified target more than 35% of the time, even after a guessing correction. Moreover, for erroneous trials, the difference between the choice of locations adjacent to the target vs. on the other side of the display was just 2% (i.e. in Experiment 1b), a difference that suggested to us that location information was not even coarse grained. To obtain a more precise estimate of location memory, Experiments 2a–c and 3 used a continuous report procedure and a three parameter mixture model to distinguish types of location errors and to measure their magnitude. We found that in cases where people identified the letter in Experiment 2a–c, they either had a precise or a very poor representation of the target’s location. Moreover, further analysis indicated that while some of the location errors were related to the location of distractors, there was also a separate proportion of such erroneous responses for which there was hardly any angular location information and this conclusion survived all attempts at guessing correction.

In Experiment 3, where targets were color marked and therefore easier to find, there was no evidence of identification without localization, which validates the paradigm’s effectiveness at finding robust location performance. At first, this result may seem contradictory to our general conclusion of the possibility of identification without localization. Yet, it is important to keep in mind that it was the single feature (i.e. color) that determined the location of the target in Experiment 3 rather than the pattern of the strokes that defined the letter. Compatible with Experiment 3 results, Carrigan et al. (2019) found no evidence for a lack of location information when they asked participants to report the location of a detected Gabor patch (i.e. a single feature stimulus) embedded in a natural scene.

Prior to discussing the results in greater depth, a brief excursion will validate the use of a mixture model for recovering parameters for paradigms with 10 stimuli. A discussion of the theoretical interpretation will continue afterwards.

### Mixture model validity testing

One might argue that increasing the number of stimuli caused the mixture model to confuse the non-target (i.e. NT) with random (pU) responses. To verify the mixture’s model ability in distinguishing NT from pU responses when 10 distractors are presented, a simulated set of response data was generated. The simulated data set was generated by re-using the set of stimulus locations from the trials with correct identity reports in Experiment 2c.

The first set of simulations generated 101 simulated data sets with the proportion of NT responses ranging from 0 to 100 (i.e. 1% increment) to examine the model’s accuracy in estimating the NT and guessing parameters. In each simulated data set, the actual locations of the stimuli on each trial was preserved but the response location was adjusted. To generate an NT response, the response location was drawn from a normal distribution of SD 18.4° (i.e. 18.4° was the SD estimated by the mixture model in Experiment 2c) centered at a randomly chosen distractor location. Simulated guess responses were generated by choosing from a uniform distribution. Target centered responses were selected from a normal distribution centered at the target with the same SD as above In these simulations, as NT was adjusted from 0–100%, the remaining responses at each iteration were divided between guessing and target responses with the ratio of 1 to 4 respectively, as this was the ratio of guessing and target probability (pU = .16, pT = .67) in Experiment 2c.

**Figure 10:**
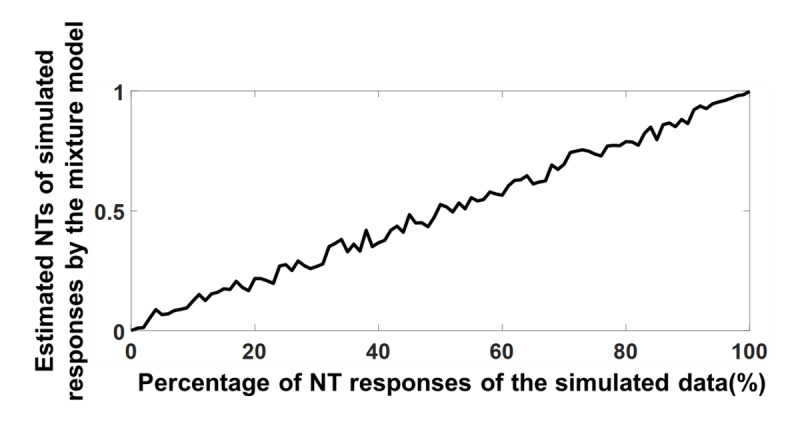
Mixture model NT testing. Proportion of estimated NT responses of the simulated data by the mixture model as a function of NT responses of the simulated data.

As shown in Figure 10, estimated and actual NTs were highly correlated, highlighting the model’s ability to recover parameters given the size of the data set used in these experiments.

Next, to better understand the model’s ability to simulate specific likelihood distributions for a data set of this size when 10 locations are used, an additional simulation was set up to mirror the estimated proportion of Guessing and NT responses from Experiment 2c. Using the same method as specified above, 16% of the responses were NT, 16% were guesses and the remaining 68% were target centered.

**Figure 11:**
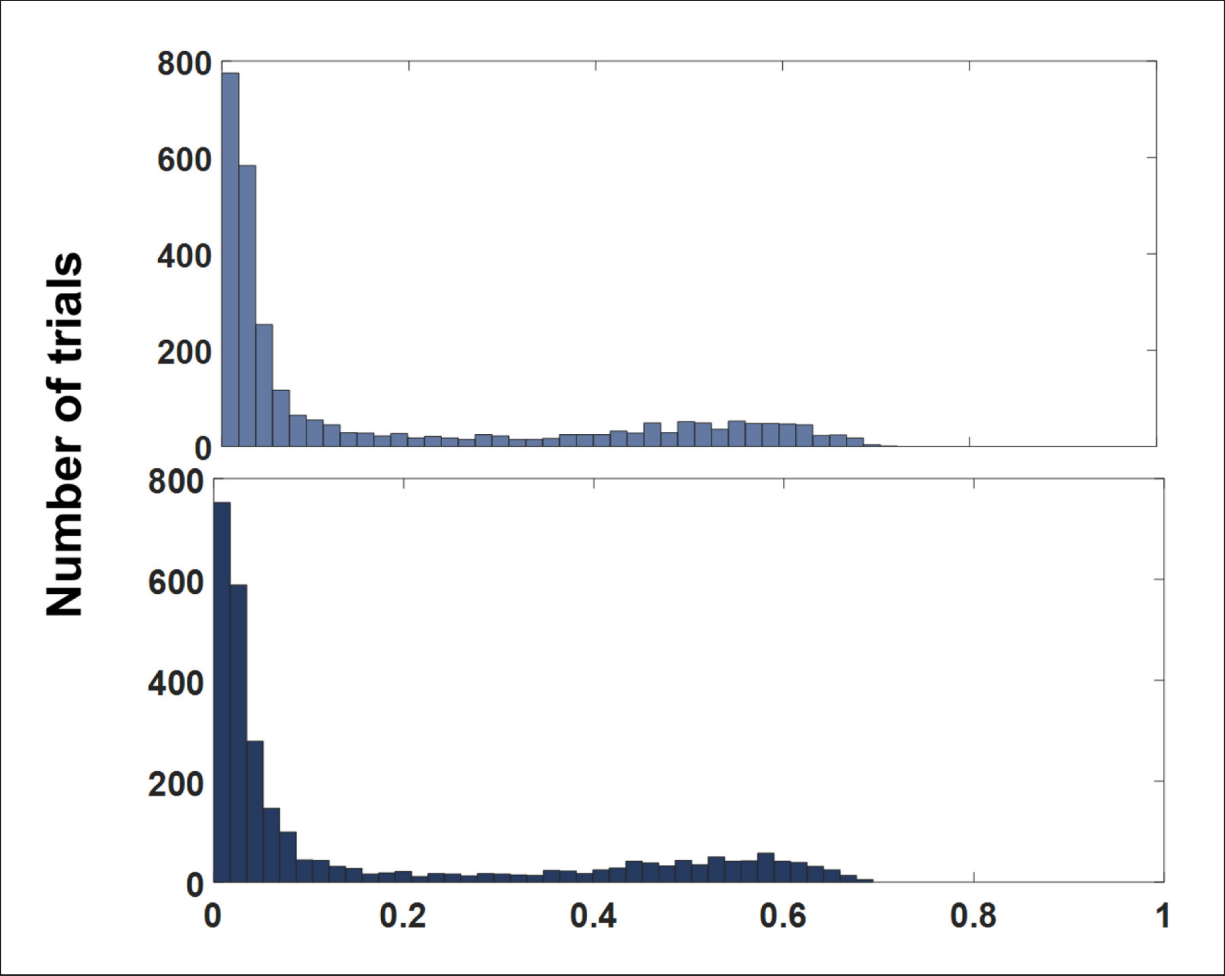
Estimated log likelihood of the NT component by the mixture model for the real data (top) and the simulated data (bottom).

Figure 11 illustrates the Log Likelihood values estimated by the mixture model for the NT component of the model for the real and the simulated data. The rightward bump in the two histograms indicates the evidence for the NT responses. The similarity of the two distributions, the top being the real data and the bottom being the simulated data where the proportion of NT responses is known to be 16%, supports the validity of the model’s estimation of likelihood functions when the number of locations is 10 and the amount of data is 2,826 trials, matching experiment 2c (i.e. Note that 2,826 was the total of correct identity trials across all subjects).

### Implications for theories of visual representation

Our finding that subjects are able to identify single visual targets without being able to remember their spatiotopic location several seconds later helps us to understand the nature of visual representations, because it reveals that location information is not fundamentally a part of the memories they create. Our results are consistent with two possibilities, either that at the moment of awareness, working memory contained very little location specific information about the target, or that the location information was so fragile as to be almost immediately forgotten. This is given the fact that subjects were attempting to remember location, since they expected to be probed about the precise location on every trial. Moreover, the poor location memory was present even on trials in which the location question occurred first, immediately after the masking display (see supplementals for more information).

One might argue that identifying the target but being unable to report its angular location in Experiments 1–2 was due to forming a verbal/phonological representation of the letter. In other words, identity was encoded into working memory as a verbal representation, whereas the location was missed due to the fact that it could not be verbalized. Forming a verbal representation of the target to facilitate memory encoding was likely to take place across all experiments, as the tasks required participants to report the target identity in every trial. However, we found improved ability to remember the location of an identified target when it was denoted by a different color even though the identity accuracy was lower (27% in Experiment 3 vs. 45.5% in Experiment 2a). Therefore, verbalizing the target could have made the identity encoding easier in both cases, but the initial target *selection* based on the color has probably given rise to a more accurate location encoding.

The lack of location information of identified letters observed in Experiments 1–2 can be understood in the context of a spatial indexing model known as FINST (Pylyshyn, 1989), where the location of an object is used preattentively to process its features, but its spatial location is not necessarily encoded into memory. In the context of the FINST framework, these results may reveal a case in which an index pointing to a letter is generated by higher level representations to identify the letter, but the rapid masking onset disrupts further processing before the spatial location is encoded into working memory. In addition, our results can be viewed within the Selective Tuning model of Tsotsos (2002). It seems that in Experiments 1 and 2, a high-level representation of a complex, familiar stimulus was activated without a corresponding link to its spatial location.^4^ This presumably happened because the ultra-rapid masking in our study interrupted the processing at earlier levels, before a binding between higher-level and lower-level representations was established. On the other hand, in Experiment 3, which had no evidence for nearly uninformed angular location reports, the recurrent binding of the target to its spatial location probably occurred through the single feature of the letter—i.e. color —instead of a high-level representation of the shape of the letter. In other words, the spatial location of the target was determined by its color, which is represented in lower levels of the visual pathway.^5^

An alternative perspective is that these results have less to say about the formation of visual percepts and more about memory. It may have been the case that the moment of awareness contained memory of location information but that representation was too fragile to be reported. If this alternative is correct, it suggests at the very least that location information was extremely weak, since there was only one target on the display to remember, and the location question followed immediately after the display in half of the trials. Additionally, forgetting the weak memory of location indicates that spatio-verbal representations that are believed to be maintained in a unified form (Prabhakaran et al., 2000), can also be maintained as separate features, because verbal representations in some trials were retained without being bound to its spatial location (Morey, 2009).

These findings resonate with the idea of VanRullen (2009) who suggests that focal attention is not necessary for binding features of familiar/natural conjunctions. On this point, VanRullen (2009) argued that two binding mechanisms coexist in the visual system. While arbitrary conjunctions (i.e. conjunctions that we do not have prior knowledge of or every day experience with, such as finding a red disc amongst red squares and green discs) require focused attention to be perceived, familiar conjunctions such as natural scenes or familiar items are recognized effortlessly without requiring focused attention. This effortless recognition takes place through hardwired brain connectivity that results from daily experiences. This account was necessary to explain the ability to discriminate complex objects without the obvious deployment of focal attention (Li, VanRullen, Koch & Petrona, 2002), a phenomenon that reveals the insufficiency of the FIT model to be generalized to all forms of visual identification. In our experiments, letters are assumed to be highly familiar items, considering that previous research on letter search tasks has also shown high efficiency in letter identification ability among other categories (i.e. digits) for college level students (Kaye et al., 1981). Given that letters are highly familiar items to our subjects, and assuming that focal attention would lead to a robust memory of location, we hypothesize that participants utilized something approaching a preattentive binding mechanism to accomplish the task.

On the other hand, identification without a clear sense of angular location is hard to reconcile with the location-specific representations found in monkey inferotemporal cortex (Dicarlo & Maunsell 2003; Beeck & Vogels, 2000). In those studies, single neurons with highly specific shape preferences also had highly specific retinotopic specificity as well. The findings here suggest that a reportable form of awareness (i.e. report accuracy clearly above chance level in a forced choice discrimination; Cheesman & Merikle, 1984) can be formed in response to a visual event without including the location-selective cells recorded in Dicarlo & Maunsell 2003. Support for the existence of such representations is found in recordings from population of neurons in inferior temporal (IT) cortex of primates that have precise identity selectivity, but substantial invariance to the object’s position (Hung et al., 2005).

Our results also have clear implications for understanding the formation of conscious mental events. In previous work, it has been suggested that visual stimuli exceeding a particular threshold of activation are able to recruit a recurrent circuit that links lower-level visual areas with a broad distribution of other cortical areas (Lamme & Roelfsema, 2000; Roelfsema & Lange, 2016). However, in cases where identity can be remembered without location, it seems that stimuli can rise to the level of awareness without being anchored to the lower level representations that exist in strongly reinotopic cortical areas. Such visually evoked representations can seemingly exist in a free-floating form that is untethered to a spatial reference. This finding does not invalidate previous theories regarding the neural correlates of awareness, but it changes our understanding of how far recurrence must penetrate into the visual hierarchy to support awareness. The implication is that information evoked by the stimulus can evoke representations that are almost purely conceptual/semantic, shedding highly specific location information in the process.

Additionally, in relation to working memory and conscious awareness, Lamme (2003) suggested that a conscious experience of a visual event, does not guarantee its storage into working memory for a later report. But an attentional deployment is required for a stable formation of that event into memory and therefore for a conscious report. Likewise, our results indicated that the memory of the percept of location was either largely absent (i.e. maybe due to lack of complete attentional deployment during the rapid presentation of stimuli), or was so weak so as to be impossible to retrieve at the time of a question even when the location question occurred directly after the masked display.

## Data Accessibility Statement

All the experiments, results and analysis are available at https://osf.io/sqn6x/.

## Additional File

The additional file for this article can be found as follows:

10.5334/joc.104.s1 Supplementary Materials. Guessing correction formulas.

## Footnotes

Note that the definitions of coarsely grained/precise responses are not precise models of the cognitive processes, but is a way of formalizing types of responses made by participants in this paradigm.

The same experiment was done with 6 stimuli as well. The pU responses and NT responses were both significantly greater than zero, however the the pU proportion was not significant after identity guessing correction. The detailed of the results and analysis can be found in supplementals (Experiment 1S).

Note that the question order was counterbalanced in all experiments. After analyzing location responses based on the order of the questions (location-first vs. identity-first) we discovered that location accuracy was worse on location-first trials in Experiments 2b and 2c, but worse on identity-first trials in Experiment 2a. These results are in Supplementary materials.

Note that we are not assuming that the high-level representation is verbal, though it may be used to generate a verbal encoding.

Another possible interpretation of the difference between the results of experiments 2 and 3 is that the higher baseline accuracy of location report and lower accuracy of identity report, led to a reduction in trials that had the conjunction of correct identity and uninformed location. Our paradigm is not able to rule out this interpretation.
