# Supplementary material for: Memories of Visual Events Can Be Formed Without Specific Spatial Coordinates
Source: J Cogn. 2020 Jun 8;3(1):13. doi: 10.5334/joc.104 (PMC7292146; doi:10.5334/joc.104)
Supplement: Supplementary Materials. — Guessing correction formulas. [file joc-3-1-104-s1.pdf]

Supplementary Materials

Guessing correction formulas

To ensure that the observed failures to report location of identified targets were not the result of lucky guesses of identity, a correction for possible guessing was applied, and the result was subtracted from the observed proportions of trials to produce estimates of the proportions of trials in which subjects correctly perceived either identity or location. Note that the essential conclusion is also supported by the uncorrected results. The guessing procedure is conservative with respect to our claim, in that it reduces the proportion of trials in which identity was reported without location.

Guessing correction for identity for all Experiments:

Step 1: The true value of identity accuracy was calculated for all experiments as follows

$$Observed\_Value = Est\_True\_Value + Chance\_level\,(1 - Est\_True\_Value) \quad (1)$$

Where O*bserved_Value* is the observed identity accuracy, *Est_True_Value* is the estimation of the percentage of trials in which the subjects successfully identified the target. *Chance_level* is the chance of responding correctly on trials in which they had not perceived the target and were guessing at random using a ratio ($^1/_{26}$). Note that subjects did not know the set of letters used in the experiment.

Step 2: Calculation of what percentage of the observed value results from lucky guesses

$$Lucky\_guesses = \frac{Observed\_Value - Est\_True\_Value}{Observed\_Value} \qquad (2)$$

Note that in **Experiments 2a, 2b, 2c and 3**, identity guessing was estimated for each subject, instead of using the grand average. This enabled us to run paired-samples t-test between estimated lucky identity and estimated uniform location responses for each subject. The results indicated significant difference between the two, suggesting that not all uniform responses can be attributed to guessing the identity, and there are some trials that people had no idea of where the target was presented.

Guessing correction for location for experiments 1a and 1b:

Since location report errors were discrete in experiments 1a and 1b rather than estimated distributions, a different procedure for corrections was used to correct for guesses for location responses where identity was successfully reported. We present this second as it is more complex and harder to understand.

Step 1: Calculate location accuracy conditional on correct report of identity

Step 2: Correct this value for location guesses

$$Obs\_Location\_Given\_Identity\_Accuracy = Est\_True\_Value\_of\_Location + \left(\frac{1}{6}\right)(1 - Est\_True\_Value\_of\_Location) \qquad (3)$$

Step 3: Calculation of identity lucky guesses

$$Obs\_Identity\_Accuracy = Est\_True\_Value + \left(\frac{1}{26}\right)(1 - Est\_True\_Value) \qquad (4)$$

$$Lucky\_identity\_guesses = \frac{Obs\_Identity\_Accuracy - Est\_True\_Value}{Obs\_Identity\_Accuracy} \qquad (5)$$

Step 4: subtract $Lucky\_identity\_guesses$ from $Location\_Given\_Identity\_Accuracy$ to estimate corrected location accuracy

$$Location\_Given\_Identity\_Accuracy\_corrected =$$
$$Obs\_Location\_Given\_Identity\_Accuracy - Lucky\_identity\_guesses \quad (6)$$

Experiment 1S

The experiment is similar to Experiment 2a, except that participants were presented with 6 stimuli instead of 8. The results have been shown in Figure 1S.

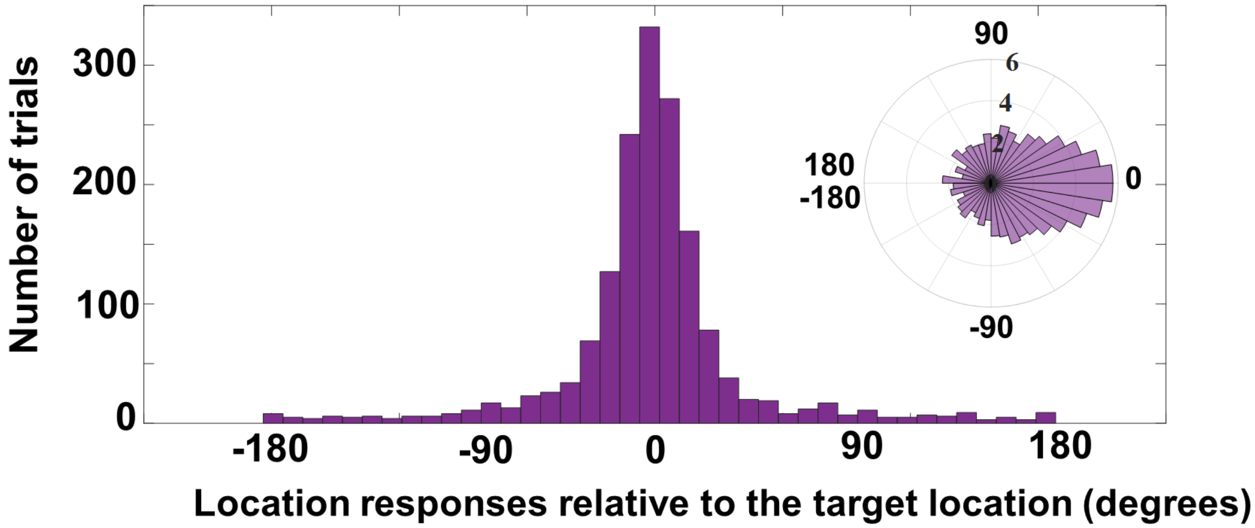

Figure 1S. Location report data of Experiment 1S for all correctly identified letters. The polar plot has been log-transformed for the ease of visualizing low-N bins.

Mixture model analysis: In the mixture model analysis for location reports for correctly identified letters with the identity accuracy of 60.5%, the K value was 15.51 (SD = 14.79

° from the 360° circle), CI[13.27, 18.13], NT was 17.07%, CI[14.05, 20.25] and the pU was 5.1%, CI[2.17, 8.31] for correctly identified letters.

Identity guesses correction: The Paired-samples t-test indicated non-significant difference between identity guesses (M = 3.72%, SD = 2.44%) and estimated pU (M = 5.35%, SD = 9.47%); $t(19) = .88$, $p = 0.39$, $BF_{10} = 0.52$. Although, it might seem that all uniform responses of location came from the identity guesses, given our subsequent Experiments with a greater number of stimuli, we argue that participants could probably remember all the 6 locations, and therefore made educated guesses when they could not remember the target location. Consider for example if participants were presented with just two items in a search array, all location reports would have been on the two locations, even if the target had not been bound to a specific location because participants might have a memory for the spatial configuration independently of the target's location. In this case, the tails of the histogram would consist of 100% Target and NT responses while pU would have been 0%.

Response order analysis:

The following table indicates identity and location responses based on the order of the questions. Comparing the pU% for identity-first and location-first trials suggests that probability of poor location responses were independent of the order of the questions in Experiments 2b and 2c.

Question order	Response accuracy/probability	Experiment 2a	Experiment 2b	Experiment 2c
**ID first**	**ID %**	50	49	51
**ID first**	**pU%**	15.04	13.5	14.14
**Location first**	**ID %**	50	51	49
**Location first**	**pU%**	4.42	16.6	18

Table 1S, **ID first** are the trials in which the identity question was asked first and **Location first** are the trials in which the location question was asked first. Note that the pU for location was estimated only for the correct identity trials.
